# GLA:D^®^ Back: implementation of group-based patient education integrated with exercises to support self-management of back pain - protocol for a hybrid effectiveness-implementation study

**DOI:** 10.1186/s12891-019-2443-1

**Published:** 2019-02-18

**Authors:** Alice Kongsted, Inge Ris, Per Kjaer, Werner Vach, Lars Morsø, Jan Hartvigsen

**Affiliations:** 1 The Nordic Institute of Chiropractic and Clinical Biomechanics, Campusvej 55, 5230 Odense M, Denmark; 2 Department of Sports Science and Clinical Biomechanics, University of Southern Denmark, Odense, Denmark; 3 Department of Applied Health Science, University College Lillebaelt, Odense, Denmark; 4 Department of Orthopaedics and Traumatology, University Hospital Basel, Basel, Switzerland; 5 Centre for Quality, Department of Regional Health Research, University of Southern Denmark, Middelfart, Denmark

**Keywords:** Back pain, Exercise therapy, Health plan implementation, Patient education, Primary health care

## Abstract

**Background:**

Reassuring patient education and exercise therapy are widely recommended interventions for back pain in clinical guidelines. However, many patients are offered non-guideline endorsed options, and strategies for effective implementation of guideline-based care have not yet been developed. This protocol outlines the evaluation of a strategy for nationwide implementation of standardised patient education and exercise therapy for people with persistent or recurrent back pain in a hybrid implementation-effectiveness design. The strategy and the evaluation were planned using the framework of the Behaviour Change Wheel.

**Methods:**

The main activity of the implementation strategy is a two-days course for physiotherapists and chiropractors in delivering patient education and exercise therapy that is aimed at supporting patient self-management. This comes with ready-to-use patient education materials and exercise programs. The clinical intervention is a group-based program consisting of two sessions of patient education and 8 weeks of supervised exercises. The program uses a cognitive-behavioural approach and the aim of the exercise component is to restore the patient’s ability and confidence to move freely. The implementation process is evaluated in a dynamic process monitoring the penetration, adoption and fidelity of the clinical intervention. The clinical intervention and potential effect mechanisms will be evaluated at the patient-level using measures of knowledge, skills, beliefs, performance, self-efficacy and success in self-management. The education of clinicians will be evaluated via clinician-level outcomes, including the Pain Attitudes and Beliefs Scale, the Practitioner Confidence Scale, and the Determinants of Implementation Behaviour Questionnaire. Effects at a national level will be investigated via data from national registries of health care utilisation and sick-leave.

**Discussion:**

This implementation-effectiveness study is designed to evaluate the process of implementing an evidence-based intervention for back pain. It will inform the development of strategies for implementing evidence-based care for musculoskeletal pain conditions, it will enhance the understanding of mechanisms for developing patient self-management skills, and it will demonstrate the outcomes that are achievable in everyday clinical practice.

**Trial registration:**

ClinicalTrials.gov NCT03570463. Registered 27 June 2018.

**Electronic supplementary material:**

The online version of this article (10.1186/s12891-019-2443-1) contains supplementary material, which is available to authorized users.

## Background

Back pain is a very common symptom in populations everywhere and is responsible for more years lived with disability worldwide than any other condition [1, 2]. The societal, health care, and economic burden associated with back pain is high and comparable to those of other prevalent, high-cost conditions such as cardiovascular disease, cancer, mental health, and autoimmune diseases [3].

Clinical guidelines for the treatment of back pain consistently recommend educating patients about back pain and its natural courses, as well as providing advice about remaining active and at work [4, 5]. In addition, they endorse supervised exercise therapy, manual therapy alone or in combination with exercises, and acupuncture, while discouraging the referral of patients to imaging and the administration of opioids, and reserving surgery for the few with very specific indications [4–6]. Guideline recommendations are, however, often not implemented because implementation of new procedures in clinical practice is challenging [7–9], guidelines are developed without any tools for implementation [10], and effective strategies for implementation have not yet been identified [11, 12]. In addition, reliance on clinical experience, perception of clinical guidelines as subjugating clinical judgment, and a limited knowledge of guideline content, are barriers to clinicians changing their behaviours [13]. Thus, care for back pain patients remains fragmented and ineffective [14, 15]. Moreover, implementation strategies in the field of back pain have generally not been developed and evaluated within a theoretical framework [11–13, 16].

In effectiveness trials, clinicians courses lasting 2 to 9 days in delivering physical and cognitive interventions translated into improved outcomes for people with back pain [17, 18]. Also, a hybrid design study on the implementation of stratified care for back pain demonstrated altered referral behaviours by family physicians and modestly improved patient outcomes with treatment delivered by 15 community-based physiotherapists trained in a course and receiving a mentoring programme [19].

One challenge in back pain treatment is that effect mechanisms of recommended interventions are poorly understood and mostly not supported by empirical evidence. Self-efficacy, pain distress and fear are involved in the transition from acute to persistent and disabling back pain [20], and pain catastrophising and perceived pain control appear to partly mediate the effects of both physical and cognitive interventions [21–23]. However, there is a need to better understand effect mechanisms if we want to identify the effective aspects of interventions and better understand why seemingly similar patients respond differently to similar treatment. In addition, it is important to note that treatment effects are reliant of the context in which care is delivered, and treatment choices for individual patients are determined by a range of factors including previous experience, expectations and patient-clinician interactions [24–27]. Therefor investigations in a real-life context is needed to understand benefits of care, individual variability in treatment response, and underlying mechanisms.

When effect mechanisms are unknown and transferability from clinical trials to practice is uncertain, the evaluation of implementing interventions in clinical practice should involve both evaluating the process of implementation as well as patient outcomes [28]. Curran et al. described studies that at the same time evaluate implementation and effectiveness at the patient-level as effectiveness-implementation hybrid designs: Type 1 hybrids focusing on effects of a clinical intervention, Type 2 with dual testing of the implementation strategy and the clinical intervention, and Type 3 that tests the implementation strategy while observing outcomes of the clinical intervention [29]. This has been suggested as a way to accelerate the translation of research findings into practice as compared with the traditional phases of up-scaling research from efficacy trials [28].

In summary, there is evidence that implementation interventions can change healthcare practitioner behaviours and potentially improve patient outcomes [12]. However, we could not find examples of studies documenting outcomes of nationwide implementation of back pain treatments, nor did we find investigations of whether implementation of recommended care can be achieved by making training generally available to back pain clinicians. Finally, there is a need for understanding how back pain treatments work in a general clinical context.

This protocol describes a hybrid effectiveness-implementation type 3 study [29], which will evaluate the national implementation of a standardised care package for people with back pain, GLA:D Back [30]. GLA:D Back will expand an existing program ‘Good Life with OsteoArthritis in Denmark’ for patients with knee or hip pain to people with back pain [31]. It aims to improve self-management in people with persistent or recurrent back pain by translating recommendations from clinical guidelines into an intervention that consists of group-based patient education and supervised exercises with data from patients enrolled in GLA:D Back included in a clinical registry that monitors patient profiles and outcomes. The main activity for implementation consists of a course for physiotherapists and chiropractors in delivering GLA:D Back and access to materials needed for the delivery of this intervention. The mode of implementation was tested in a feasibility study with physiotherapists and chiropractors from nine clinics that all managed to implement the intervention following the course. These pilot clinics enrolled 89 patients in the GLA:D Back programme and 161 in two comparison groups as part of the feasibility testing. A mixed methods evaluation showed good promise for adoption of the clinical intervention after clinicians’ course participation, for the data collection and for positive outcomes with the GLA:D Back intervention [32].

This protocol describes the methods for evaluation of the initial roll-out of GLA:D Back. This includes evaluating the clinical intervention targeted at patients, the educational intervention targeted at clinicians, and the national implementation.

The study objectives for the evaluation of the clinical intervention are:

- To describe changes in patient outcomes after participation in GLA:D Back and evaluate if these are of a magnitude comparable with those observed in RCTs on combined patient education and exercise interventions for persistent and recurrent back pain,
- To determine to what extent patterns of health care utilisation and sick leave change in individuals from one year before participation in GLA:D Back to one year after,
- To determine if patient outcomes are associated with clinicians’ treatment orientation and confidence,
- To identify subgroups of patients who do not benefit sufficiently from the GLA:D Back clinical intervention, and
- To investigate potential mechanisms of change in patient outcomes.

With respect to within subject changes we hypothesise that the clinical intervention is as effective as previously observed changes in RCTs on similar interventions.

The study objectives for the evaluation of the clinician educational intervention are:

- To monitor outcomes of learning activities and adapt the delivery of the clinician course accordingly,
- To determine if there is a change in treatment orientation from a biomedical to a more behavioural orientation and an increase in clinicians’ confidence in managing people with back pain following the GLA:D Back course,
- To identify clinician factors related to treatment orientation and confidence in managing back pain, and
- To determine if treatment orientation and clinicians’ confidence are associated with clinical behaviours.

We hypothesize that the educational intervention is effective in preparing the clinicians to deliver the clinical intervention as intended.

The study objectives for the evaluation of the national implementation are:

- To monitor the promotion of GLA:D Back courses and adapt the promotion strategy when prespecified criteria are not met,
- To describe the penetration, adoption, and fidelity of GLA:D Back including the degree of uniformity across clinicians, clinics, and regions,
- To identify individual and organisational determinants of adoption and fidelity, and
- To evaluate the effects of the implementation on referrals to imaging and secondary care for back pain, opioid prescription, and sick leave rates at the national level.

We hypothesize that in the long run the implementation will imply reductions in referrals to imaging and secondary care and in opioid prescriptions for back pain at the national level.

## Methods

This protocol is presented in accordance with the Standards for Reporting Implementation Studies (StaRI) Statement [33], and the educational intervention for clinicians in agreement with the Guideline for Reporting Evidence-based practice Educational interventions and Teaching (GREET) [34]. The study was registered in Clinicaltrials.gov May 2018 (ID: DPA 2015–57-0008 SDU 17/30591).

### Theoretical framework

**Fig. 1:**
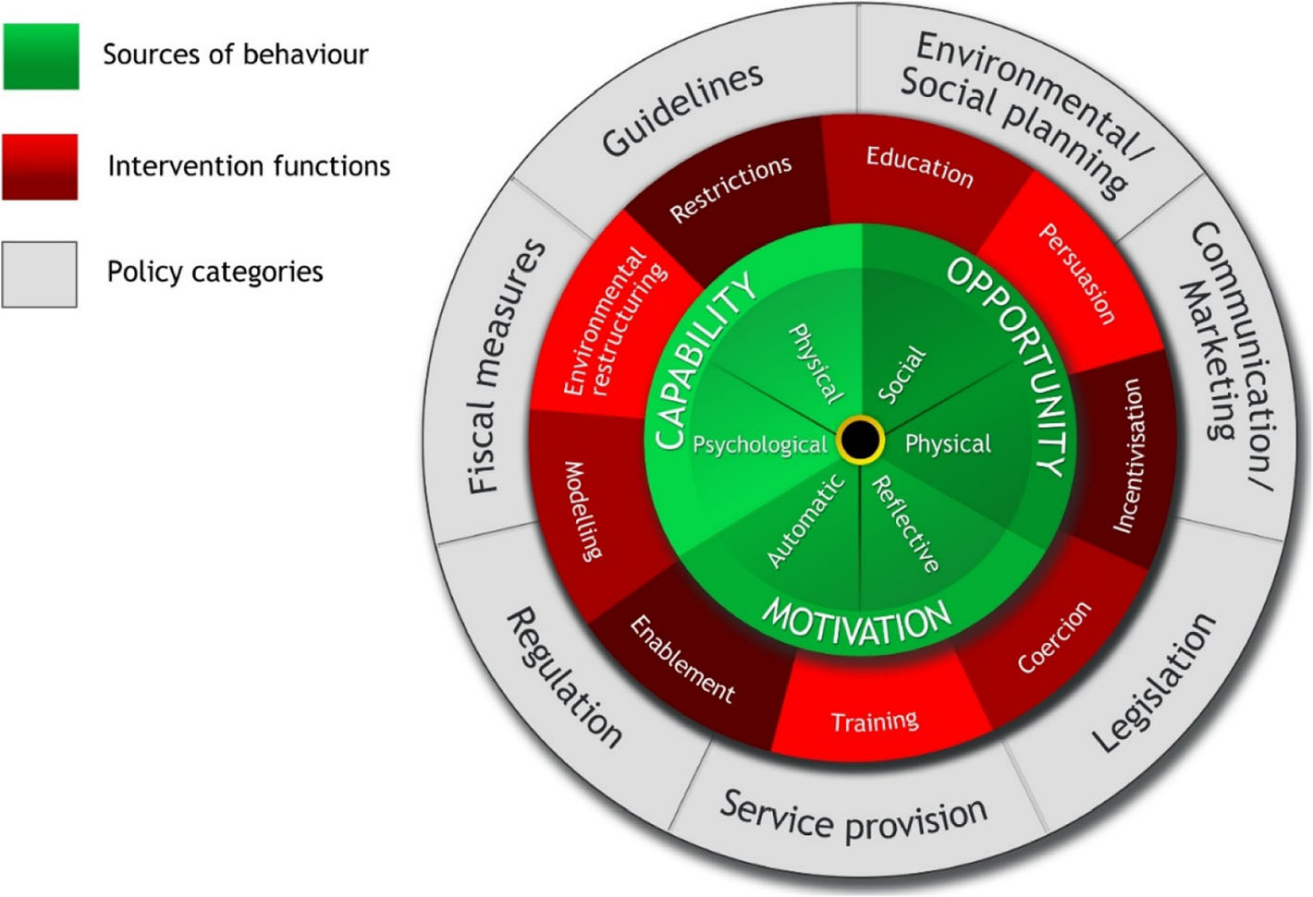
The Behaviour Change Wheel. Susan Michie et al. *Implementation Science* 2011 6:42 [35]

We used The Behaviour Change Wheel as a theoretical framework for aligning the objectives, strategies and evaluation of implementation. It was developed as a framework for characterising interventions and policies intended to change behaviour [35]. It is based on elements from 19 theoretical frameworks of behaviour change and incorporates domains from the Theoretical Domains Framework. The Behavior Change Wheel links types of interventions to mechanisms of behaviour change centred on three core elements: ‘Capability’, ‘Opportunity’, and ‘Motivation’ the (the COM-B model), and provides a framework for considering both organisational and individual levels of implementation (Fig. 1).

### Context

Denmark is a European country with around 5.7 million inhabitants. It spends approximately 10% of its gross domestic product on healthcare, 10–16% of which is spent on primary care [36]. Danish primary care is administered by five regions and 98 municipalities. The regions are responsible for most of the primary care services including general practitioners (GPs), physiotherapists, and chiropractors whereas the municipalities are responsible for rehabilitation.

GPs, physiotherapists and chiropractors in primary care are self-employed and most have a contract with the state-funded universal health insurance that is negotiated collectively by their professional organisations. In 2017, approximately 4100 GPs, 2850 physiotherapists and 410 chiropractors were covered by these agreements [37]. Visits to GPs are fully reimbursed with no out-of-pocket expenses incurred by the patient, whereas between 60 and 80% of the fee for physiotherapy and chiropractor services are paid for by the patient directly or through a private insurance company. Costs associated with physiotherapy are reimbursed by the regions only if patients are referred by a GP, whereas reimbursement for chiropractic costs is independent of referral. Both chiropractors and physiotherapists can offer services outside of these contracts, but in these situations there is no reimbursement or negotiated prices. Approximately 25% of chiropractic clinics have at least one physiotherapist employed as a member of the staff [38].

In addition to the universal health care coverage, approximately 1.9 million Danes currently have private health insurance fully or partially covering out-of-pocket expenses for services from physiotherapists, chiropractors, psychologists and other providers.

GLA:D^®^ for patients with knee and hip pain was established as a non-profit initiative by the University of Southern Denmark (SDU) in 2013 [31]. The GLA:D trademark is registered by SDU and is reserved for initiatives that train clinicians in delivering guideline-supported interventions for musculoskeletal disorders with outcomes recorded and monitored in a registry approved by SDU. After 5 years, more than 1000 physiotherapists from primary care and municipalities have participated in a GLA:D course and 32,600 patients have been recorded in the GLA:D registry, up until May 2018. As such, GLA:D is the only Danish example of a well-described clinical intervention for musculoskeletal conditions that is widely available and for which there is a systematic registration of patient-reported and clinical outcomes. Since 2016, one region has provided additional reimbursements for GLA:D knee and hip, and in that region it is now a requirement that patients have participated in a course of structured, supervised exercises prior to being seen by a surgeon in a public hospital. To date, there has not been any systematic evaluation of the implementation of GLA:D knee and hip.

### Interventions

#### The GLA:D Back clinical intervention

**Fig. 2:**
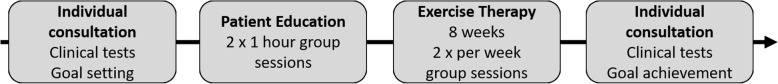
Outline of the clinical intervention

This clinical intervention is intended for people with persistent or recurrent low back pain (LBP) and in need of improved self-management. The content and intervention mapping are presented in detail elsewhere [30]. Briefly, GLA:D Back consists of an individual session of clinical testing and goal-setting at the beginning and end of the program, two one-hour group sessions of patient education, and 8 weeks of twice-weekly one-hour supervised exercises sessions (Fig. 2).

**Fig. 3:**
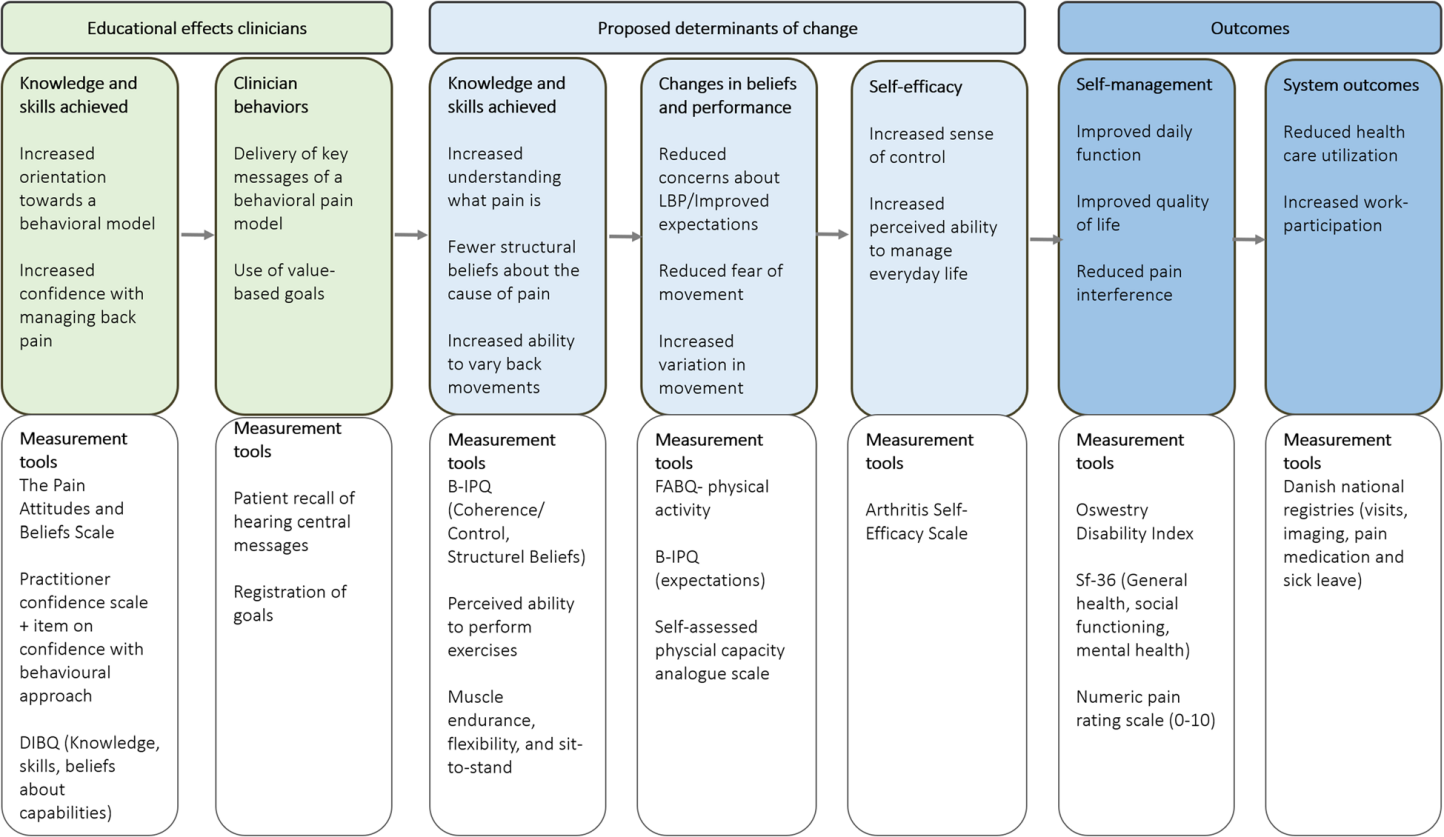
The theoretical model of change at the clinician level and the patient-level

The overall aim of GLA:D Back is to improve the participant’s ability to self-manage and the content is based on a cognitive behavioural approach aimed at supporting pain self-efficacy. Theoretically, the proposed effect mechanisms are that the knowledge and skills achieved during participation in the program translate into changed believes and performance, which in turn will improve self-efficacy, decrease disability, increase quality of life, and ultimately reduce health care utilisation and sick leave (Fig. 3).

**Fig. 4:**
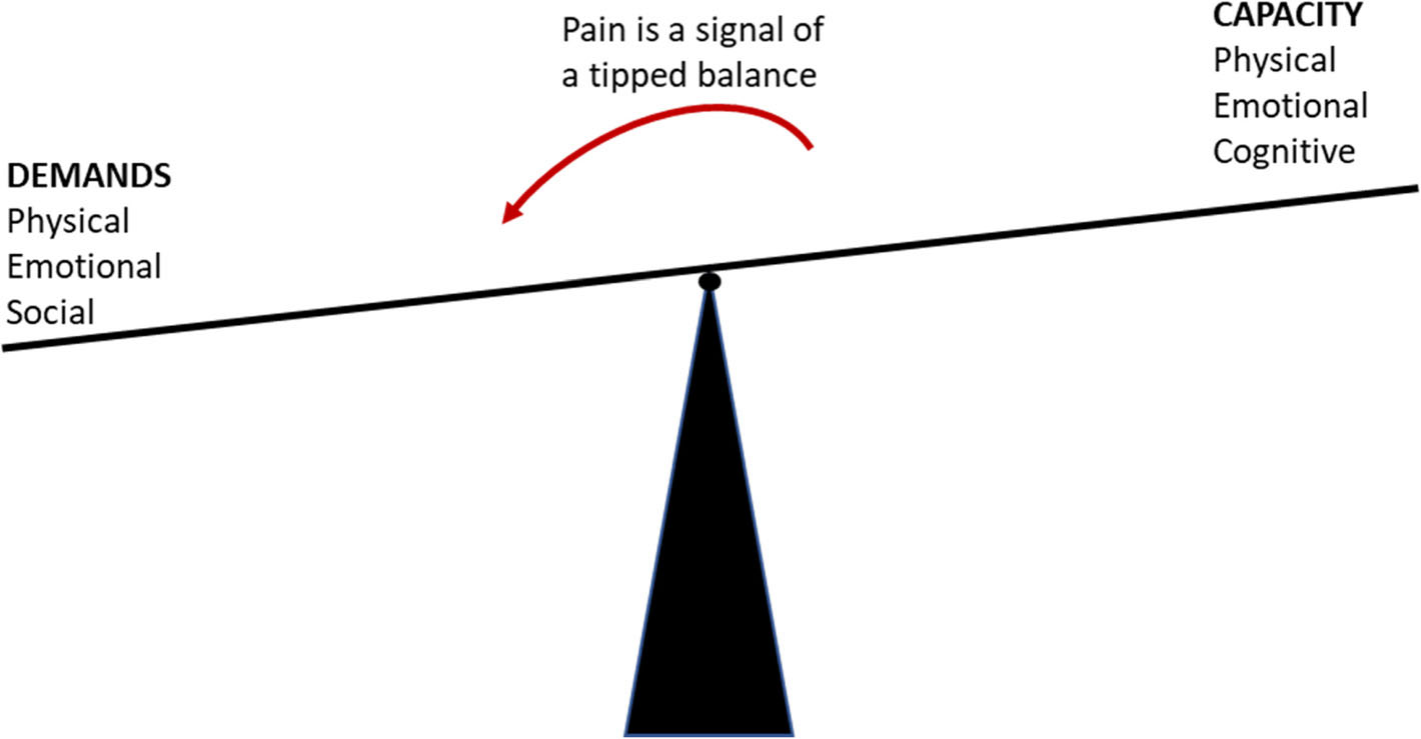
Illustration from the patient education explaining that pain is a result of your demands (physical, emotional and social) exceeding your capacity (physical, emotional, and cognitive)

**Fig. 5:**
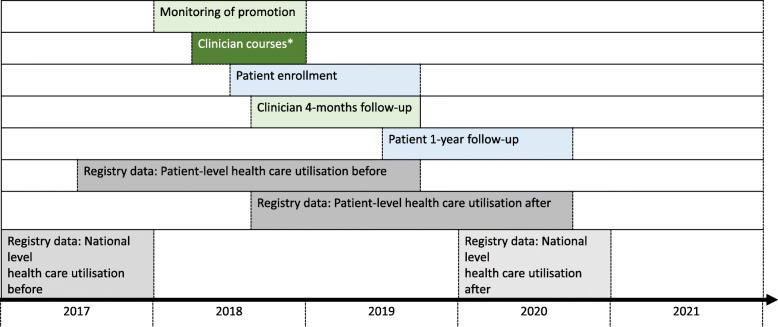
Study timeline. * Clinician data collected before and after the course

Clinicians can adapt GLA:D Back to their particular setting and to the needs of individual patients, however, the following core parts cannot be altered: 1) an individual session at the beginning and the end of the program with goal-setting and physical tests, 2) 2 h of patient education and 16 sessions of supervised exercises over 8 to 10 weeks, 3) key messages stating that pain is not a sign of danger, 4) an explanation of back pain using a behavioural model of (im) balance between demands and capacity rather than emphasising tissue damage (Fig. 4), 5) guidance for patients in exploring movement rather than being taught to perform exercises in one ‘correct’ manner, and 6) entry of data into the clinical registry. Refinements of the content of the patient education or exercise program will be made continuously by the research group without violating these principles.

#### The clinician educational intervention

Clinicians are trained in a two-day course (7 h each day) delivered by the developers of the program (AK, IR, PK, JH) and by employed expert clinicians. The course program can be seen in Additional file 1.

The primary aim of the course is to motivate clinicians to adapt a behavioural rather than a biomedical/structural treatment orientation and to provide tools that support the implementation of the GLA:D Back program. The GLA:D Back course was developed using the COM-B model in order to supports clinicians’ behaviour change by improving their capability, motivation and opportunity to change. The learning goals are pursued by using different educational elements including education through lecturing, persuasion and modelling by examples, in addition to skills training (Table 1).

**Table 1:** Learning goals and learning activities of the clinician course based on the COM-B model

COM-B TDF domains	Learning goals	Learning Activity (Interventions)	Evaluation tools
Motivation			
Social/professional role and identity	Clinicians perceive their role in ensuring high-quality care as important	A ‘state-of-the-art’ lecture including an overview of the evidence on the burden of back pain, the prognosis of back pain and the call for non-pharmacological, non-surgical interventions in clinical guidelines (Ed)	DIBQ (Social influences)
Beliefs about capabilities	Clinicians feel confident that they are able to deliver GLA:D Back	Reinforcement that most of the skills needed are pre-exiting among educated clinicians (Ed) Group exercises focused on practising delivery of key messages of the patient education. Participants take turns in playing the roles of the teaching clinician and mentor. A scoring guide (rubric) is used to facilitate feed-back (En, T) Clinicians receive the patient education content as a slideshow with manuscript, exercise programs and handouts containing examples of language to be used in delivering key messages (En)	Practitioner Confidence Scale DIBQ (Skills, Knowledge, Beliefs of capabilities)
Optimism	Clinicians believe that GLA:D Back will add value	A recording of a patient interview providing a patient’s views on what was gained from taking part in GLA:D Back (P) Posters placed at the course venue with quotes from clinicians and patients who have experienced GLA:D Back (P)	DIBQ (Beliefs about consequences)
Beliefs about consequences	Clinicians agree with a need for standardised evidence-based back pain care Clinicians have positive attitudes towards a behavioural model for back pain treatment	Presentation of facts about the adoption of GLA:D knee and hip and the achieved political agreements for integrating GLA:D in disease management programs with reduced out-of-pocket expenses (Ed, I, M) Lecture about ‘state-of-the-art’, pain mechanisms, the evidence-base for GLA:D Back and the hypothesised mechanisms of action (Ed) Presentation of patient outcomes and statements from clinicians about experiences with GLA:D Back (P, I) A recording of a patient interview providing a patient’s views on what was gained from taking part in GLA:D Back (P)	DIBQ (Beliefs about consequences, Patients, Innovation) The Pain Attitudes and Beliefs Scale
Intentions	Clinicians intend to offer GLA:D Back in their clinics	Facilitated group discussion about practical organisation with examples of solutions from test-sites (M) Participants documenting a plan on when, where, who, and how to initiate the GLA:D Back-program in their clinic (P, En)	DIBQ (Behavioural regulation, Intentions) Intended time point for starting the first GLA:D Back-group
Reinforcement	Clinicians are aware that GLA:D for hip and knee has been well received by physiotherapists, patients, general practitioners and politicians	Presentation of facts about the spread of GLA:D knee and hip, the patient outcomes, and the achieved political agreements for integrating GLA:D in disease management programs with reduced out-of-pocket expenses (Ed, I, M)	Not measured
Emotion	Clinicians appreciate the value of being a GLA:D Back instructor	A recording of a patient interview providing a patient’s views on what was gained from taking part in GLA:D Back (I)	Not measured
Capability			
Knowledge	Clinicians know current recommendations for treatment of back pain Clinicians know the GLA:D Back approach to support patients’ self-management Clinicians know why SMART value-based goals are used	A ‘state-of-the-art’ lecture including an overview of the content of clinical guidelines on treatment (Ed) A lecture on the patient education that links the content of GLAD Back to self-efficacy (Ed) A lecture about value-based goals, demonstration of web-tool for goal registration, examples from the pilot (Ed, T, I)	DIBQ: Knowledge
Skills	Clinicians can identify relevant patients for the program Clinicians can deliver the key messages of the patient education Clinicians can support patients in identifying goals Clinicians know how to perform the exercises in the program Clinicians can apply knowledge of the non-structural pain model in the supervision of exercises Clinicians can supervise exercises in a manner that helps patients explore movement Clinicians can enter data in the database	Definition of the target group for the clinical intervention (Ed) Provision of examples of explaining back pain using the educational material (T) Provision of examples of questioning technique for identifying goals (T) Instructions on how to perform Texercises in the program (T) Group exercises focused on practising the delivery of central messages of the patient education. Participants take turns in playing the roles of the clinician teaching and the patients with diverse issues and worries (En) Exercise based on video cases to practice how messages from the patient education are used in the instruction of exercises. A scoring guide (rubric) is used to facilitate the evaluation of cases (En) A lecture on using the digital platform to enter data (Ed)	Enrolled patients report long-lasting or recurrent LBP and have similar profiles across clinicians Delivery of central messages (patient reported) Use of individual goals registration in the registry DIBQ: Skills, beliefs about capabilities Clinicians use the register
Behavioural regulation (action planning, breaking habits)	Clinicians know how to get started with GLA:D Back	Clinicians documenting a plan for when, where and how to start their first GLA:D Back-group and discuss their plan in groups (En) A lecture on using the digital platform to enter data (Ed) Following the course, access to educational materials, exercise programs and instructions for clinical tests are available online. Clinicians have access to the technical support and to the research team for guidance when questions arise (En)	Patients are enrolled in and complete the GLAD programme
Opportunity			
Environmental context and resources	Clinicians know that the programme does not require high-tech equipment	Emphasising the use of low-tech equipment while delivering the GLA:D programme throughout the course and demonstrating this when teaching the exercises (E, En)	DIBQ (knowledge)
	Clinicians see how the programme can fit into existing routines and payment structures	Workshop where clinicians share experiences with implementing back programs and are able to ask questions of expert clinician	DIBQ (knowledge)

An overview of the learning goals, course activities and clinician-level outcomes in GLA:D Back intended to address elements that, within the Theory of Planned Behaviour, affect clinicians’ intentions to change and their actual change in behaviour. The interventions of Education, Training, Enablement and Incentivisation are defined as part of the Behavioural Change Wheel

*Ed* Education – Increasing knowledge and understanding, *P* Persuasion – Inducing feelings to stimulate action, *T* Training – Communicating skills, *En* Enablement –Reducing barriers to increase capability, *I* Incentivisation - Creating expectation of reward, *M* Modelling – Exemplifying to aspire or imitate, *E* Environmental restructuring – changing context (physical/social), *DIBQ* Determinants of Implementation Behaviour Questionnaire

First, participants acquire an understanding of the content of the clinical intervention and the arguments for standardised care. They are presented with an overview of current evidence about the burden of LBP, evidence supporting persistent LBP as a non-injury pain condition, a brief introduction to pain modulation and the cognitive behavioural model [39], and a summary of the evidence base for GLA:D Back. Clinicians are informed that GLA:D Back was developed for people with persistent or recurrent non-specific LBP who have a need for improved self-management, and the decision as to whether or not a patient enters the program is left up to their clinical judgement and the patient’s motivation.

Second, the main body of the course is aimed at developing participants’ ability to deliver the GLA:D Back clinical intervention as a way of addressing pain cognitions and behaviours by introducing all elements of the clinical intervention: goal-setting, clinical tests, patient education and supervised exercises. Goal-setting is introduced by presenting the SMART-model (Specific, Measurable, Acceptable, Realistic, and Time bound goals) [40]. Role plays to practice the use of explanatory models and skills training are used to become familiar with pain education, physical tests and exercises. Using some of the slides from the patient education material, participants work in groups taking turns to deliver key messages from the pain education to each other as they would in a real patient session, evaluated using peer feedback. The clinical tests and exercises are introduced in a practical session where tests are carried out on a colleague, and exercises are performed by the participants while examples of variations are presented. The supervision of exercises is practised in a session based on video cases and centred on the messages (spoken and unspoken) that can be delivered while supervising patients in the clinic. Key messages from the patient education should be reinforced during the instruction of exercises, for example, when explaining to patients how to cope with pain provocation during exercises rather than withdrawing from the activity. Finally, participants are introduced to the clinical registry used for data entry. At the end of Day 2, clinicians plan how and when to start GLA:D Back in their clinics. They are encouraged to pursue implementation by referring to the success of the GLA:D Knee/Hip model and the experience with GLA:D Back from pilot testing, as well as by emphasising the importance of physiotherapists and chiropractors taking on the role of ensuring access to evidence-based care for back pain.

Courses may be refined during the process of implementation, however, the core parts that are not subjects to change are: 1) familiarising participants with the key messages of the clinical intervention, 2) the introduction to the clinical registry, and 3) that the clinicians practice the delivery of patient education and the instruction of exercises during the course.

#### The national implementation

The national implementation will be driven by clinicians who have attended the course and are trained in GLA:D Back. Therefore, the first step was to generate awareness of the development of GLA:D Back in professional journals, at seminars and meetings, and by mentioning the new initiative at the GLA:D Knee/Hip courses. Then, in August 2017, nine clinics (31 clinicians) participated in piloting the GLA:D Back program and took their own initiatives to promote their participation via websites, social media and contact with GPs.

In February 2018, when course registration opened for all interested chiropractors and physiotherapists, the Danish Physiotherapy Association and the Danish Chiropractors’ Associations and some physiotherapy special interest groups were asked to advertise the program to their members. The GLA:D Back website, and an invitation to register, were also promoted by the research team on Facebook, LinkedIn and Twitter and to clinicians who use the GLA:D register for knee/hip patients when they logged on to the registry (Table 2). Course participation is at a cost of approximately €550 (2018).

**Table 2:** Promotion activities and pre-defined minimum standards for the planned activities and for reach

Planned promotion activity	Interval	Minimum standard
Article on the Danish Physiotherapists’ web site	Completed 2017	NA
Poster presentation Danish Chiropractors’ Association meeting	Completed 2017	NA
Series of three letters on standardised care in a magazine for members of Danish Chiropractors’ Association	Completed 2017	NA
The Danish Chiropractic Association (DCA) informs members that course registration is open	Once before registration opens	One newsletter e-mailed to members of the DCA News posted on the DCA’s website
The Danish Physiotherapy Association informs members that course registration is open	Once before registration opens	One newsletter e-mailed to members of the Association News posted on the Association’s website
Information about the GLA:D Back courses at websites of physiotherapy special interest groups - Musculoskeletal physiotherapy - Sports physiotherapy - Physiotherapists in private practice - Physiotherapists without a contract with the board of wages and fees	Once before first course registration closes	One of the listed groups posts the information
Direct e-mail to the five regions’ private practice consultants from chiropractic, physiotherapy and general practice	Once when information about opening the registration is known	Mail send before registration opens
Information to clinicians who deliver GLA:D for knee/hip patients on the front page of the knee/hip clinical registry	When registration is open	Information posted one time before opening
Promotion of GLA:D Back via social media (Twitter, Facebook, ResearchGate, LinkedIn)	When GLA:D Back related external activities (courses, talks) and publications occur	One posting per month during 2018
Workshop at the yearly seminar for general practitioners “Lægedage”	November 2018 + November 2019	One workshop accepted
Clinicians who participated in the course and wanted to deliver the intervention listed at the GLA:D Back website	After courses are conducted for a region	Updated within 2 weeks of the last course for a region
Target	Group definition	Minimum standard
Number of clinicians educated in 2018	The Capital Region of Denmark	60
	Region Zealand	60
	Region of Southern Denmark	60
	Central Denmark Region	60
	The North Denmark Region	60
	Total	300
Years of clinical experience (min. proportion)	0–5	10%
	> 15	10%
Sex	Proportion males	Min 33% Max 66%
Work place and role (min. proportion)	Municipality	5%
	Private clinics	60%
	Clinic owner	5%
	Employed clinician	50%
Profession (minimum proportion)	Physiotherapist	70%
	Chiropractor	10%

Clinicians who participate in the course and decide to deliver GLA:D Back at their clinic are listed as certified clinicians on the GLA:D Back website (http://gladryg.sdu.dk/). Because GLA:D is a registered trademark, only clinicians trained at SDU can use the brand, and sustained certification comes with the requirement to enter all appropriate patient information into the clinical registry. Updates and re-certification are planned for the future.

To support the implementation after the course, clinicians are given access to detailed patient educational materials, exercise programs, and written suggestions of language that can be used in the supervision of exercises after the course. They also are given GLA:D Back logo t-shirts and posters for the clinic with key messages from the patient education and an overview of the exercises [30]. To assist a uniform promotion of the program, clinicians get information leaflets directed at patients and at GPs that can be used with their individual clinic names and logos.

There are no strict patient inclusion criteria, and the decision to enrol a patient in GLA:D Back is at the discretion of the clinician in a dialogue with the patient, when clinicians judge that the patient would benefit from improved self-management skills. Patients can be enrolled at the first visit or later depending on whether individual treatment sessions are deemed necessary prior to starting GLA:D Back. When patients begin the program their contact information is entered in the clinical registry, which in turn activates links to patient questionnaires that are sent automatically on the day of registration and again after 3, 6 and 12 months. Clinicians enter results of physical tests and patients’ individual goals in the registry before patients start the GLA:D Back intervention and when the program ends. The price and out-of-pocket expenses for the GLA:D Back intervention can vary between clinics and types of insurance.

Consultants for physiotherapy, chiropractic, and family medicine working with primary care in the five administrative regions were invited to an information meeting about GLA:D Back before course registration was opened (January 2018). Family physicians will be informed through professional journals and seminars.

### Effectiveness and process evaluations

#### Data collection

Data sources will include: 1) data obtained from clinicians when they register for a GLA:D Back course, 2) clinician-completed surveys before and directly after the course and after 5- months, 3) observations of patient education and exercise group sessions in selected clinics, 4) patient physical tests at baseline and at the end of treatment, 5) patient-completed surveys at baseline, 3-months, 6-months and 12-months, 6) patient interviews, and 7) information from Danish registries (Fig. 5). Patient and clinician data are collected electronically using the REDCap software (Vanderbilt University) provided and supported by the Odense Patient data Explorative Network (OPEN) [41].

#### Evaluation of the clinical intervention

The clinical intervention will be evaluated based on data about the patients who enrol in the GLA:D Back program, i.e. those patients who are registered in the GLA:D Back registry.

Due to the lack of a control group we will compare mean changes in these patients with those observed in the intervention groups of trials that have demonstrated positive effects of patient education and exercise therapy. We will look for trials with little risk of bias that included patient populations comparable with those enrolled in GLA:D Back.

Health care utilisation and sick leave will be investigated using national registry data to compare individual patterns of health care utilisation (primary care visits, hospital visits due to LBP, imaging for LBP, prescriptions for pain medication) and sick leave from 1 year before participation in GLA:D Back to 1 year after participation (Fig. 5).

Clinicians’ beliefs about back pain and confidence in managing patients with LBP (described below) and their baseline characteristics will be investigated as potential predictors of patient outcomes. Potential mechanisms of change in patients will be investigated by testing the mediating pathways outlined in the theoretical model (Fig. 3). We will specifically test the extent to which improved illness beliefs, increased perceived ability to perform exercises, and improvements on physical tests may explain a reduction in fear avoidance, improvement in expectations and increased self-assessed physical capacity during the 3 months after inclusion and during the following 3 months (3- to 6-months follow up). We will then test to what extent reduced fear avoidance beliefs, improved expectations and increased physical capacity during the initial 3 months relate to an increase in self-efficacy during the same period and to improved self-efficacy after 6 and 12 months. Next, we will investigate the relationship between increased self-efficacy and outcomes of self-management success, and whether self-efficacy is a mediator between beliefs and performance and success in self-management. Lastly, we will test to what extent improvements in disability, quality of life and pain after 6 months predict health care utilisation and sick leave after 12 months, to what extent improved self-efficacy influences these outcomes, and if this is fully or partly explained by improvements in self-management.

Patients’ perceptions related to developing pain self-efficacy and ability to self-manage will be explored using individual interviews that address back beliefs, perceived ability to manage LBP, and what aspects of the LBP experience need to change during and after participation for patients to consider the intervention beneficial.

#### Evaluation of the clinician educational intervention

All clinicians participating in a course will be included in this evaluation. The course content and delivery will be evaluated via observations by an expert in medical education to confirm that the planned learning activities are delivered. Outcomes of learning activities (Table 1) will be compared between courses and any trends in change over time described.

Evaluations of clinician-level outcomes will be investigated in an observational longitudinal design with clinician-reported data collected before the clinician training course, immediately after the course and 5 months later, and with patient-reported information on delivery collected at their three-months follow up. Changes over time in treatment orientation and confidence in managing patients with LBP will be described, and potential relationships between clinician characteristics, treatment orientation and confidence will be determined.

#### Evaluation of the national implementation

The implementation process will be evaluated, starting with the promotion of courses for clinicians, the degree of course participation, the adoption and fidelity of the implementation in the clinics, through to the potential impact on health care utilisation at a national level. An evaluation of sustained implementation is not part of this protocol. Elements of the process evaluation are listed in Table 3.

**Table 3:** Overview of the aspects of the national implementation that are evaluated and the related measurement

Process	Definition/Measurement	Data Source
*Spread (promotion of GLA:D Back)* Newsletter from professional organisations Information to GLA:D Knee/Hip deliverers Description of GLA:D Back in magazine for general practitioners Information on the GLA:D Back web site Use of social media	The extent to which the intended promotion activities are delivered (Table 2)	Web sites, social media, e-mails
*Clinician reach*	The proportion of chiropractors and physiotherapists, on a contract with the universal health insurance, participating in GLA:D Back courses. Profile of enrolled clinicians.	Course registration
*Penetration and adoption (extent of implementation)*	Geographical penetration measured as the number of municipalities with at least one GLA:D Back deliverer. Rate of adoption will be measured as the proportion of clinics with trained clinicians offering GLA:D Back (defined as having entered at least five patients in the clinical registry) within 6 months of course participation. The number of patients starting GLA:D Back	GLA:D Back registry
*Fidelity (quality of implementation)*	The extent to which the core elements of the clinical intervention are delivered	Observations in selected clinics Patient 4 month surveys
*Determinants of implementation*	The domains of knowledge, skills, beliefs about capability, beliefs about consequences, innovation, patients, intentions, organisation, social influences, social context, and behavioural regulation are captured by the Determinants of Implementation Questionnaire (DIBQ) [16].	Clinician surveys
*Patient participation*	Proportion of patients enrolled in GLA:D Back that complete the program Profile of enrolled patients and of completers/non-completers Reasons that patients do not want to participate	Patient surveys in the GLA:D Back Registry Clinician surveys

Promotion of courses and pre-defined minimum standards for each element of the promotion strategy will be monitored during the roll-out (Table 2). A research team, not otherwise involved in GLA:D Back, will conduct the monitoring and provides feedback to the GLA:D Back project group at regular intervals, which in turn will determine the actions required when standards are not met. For example, if one region has less than 60 clinicians registered, this will be fed back to the project group with information on any particular clinician groups (e.g. related to professional background or clinical experience) that appears not to have been reached so that actions can then be taken to reach that specific group(s).

The adoption of GLA:D Back will be investigated by measuring the extent to which clinicians who have participated in the GLA:D Back course conduct the program in their clinics. Individual clinician factors and organisational factors will be investigated as determinants of adoption.

Fidelity will be investigated quantitatively by asking about treatment content and recall of key messages via patient questionnaires, and by ensuring that patients’ individual goals are registered. The quality of care in terms of delivering the key elements of the intervention will be further explored by video-based observations and from patient interviews that will provide a patient perspective on the content of care.

The profile of patients enrolled in the GLA:D Back programme will be compared between clinics and administrative regions to investigate if clinicians seem to agree on the target group as well as detect if regional politics or planning affects patient selection. Also, the proportion and profile of patients enrolled who do not complete the program will be compared across clinician profiles and regions. Finally, we will ask clinicians about their experience with patients being motivated to enrol in the program and the reasons why patients considered suitable candidates for the program declined participation.

The frequency of referrals to imaging and to secondary care for back pain, opioid prescription and long-term sick leave related to LBP at the national level will be compared between 2016 and 17 before implementation starts and during 2020–21, i.e. commencing one year after all Danish regions have had clinicians educated. Comparisons will be made at the overall population level as well as for populations with a history of LBP identified from sources such as The Danish National Health Survey.

### Outcome measures

Outcomes at the clinician level and patient level will be matched to the theoretical model of change in Fig. 3.

#### Outcomes of the clinical intervention

An overview of the study measures is provided in Table 4.

**Table 4:** Overview of study measurements related to the clinical intervention

Construct	Instrument	Patient reported				Clinician reported		National registries
		Baseline	3-months	6-months	12-months	Pre- intervention	Post- intervention	
Demographics	Age, sex, height, weight, education	x						
Work situation	Job type, working hours	x						
Risk profile	The START Back screening tool	x						
LBP history	Pain duration (0–2 weeks; 2–4 weeks; 4–12 weeks; 3–12 months; > 1 year) Pain trajectory last 12 months (one of seven patterns or ‘I don’t recognise any of the patterns)	x						
Comorbid pain	Pain in any of nine regions on body chart in the last 2 weeks	x			x			
Comorbidity	Any chronic disease (yes/no) on a list of 15 conditions					x		
Illness perceptions	The Brief Illness Perceptions Questionnaire [42, 43]	x	x	x	x			
Ability to move with variation	How confident are you in performing exercises in a beneficial way?’ (0–10 scale from ‘not confident at all’ to ‘absolutely confident’)	x	x	x	x			
Physical back performance	Standing forward bending [46] The Ito back extensor endurance test [49, 50] Trunk flexor endurance test [50, 51] Sit-to-stand test					x	x	
Perceived physical fitness	Self-assessed physical capacity [57]	x	x	x	x			
Fear of movement	Fear Avoidance Beliefs Questionnaire [54, 56]	x	x	x	x			
Self-efficacy	The Arthritis Self-efficacy Scale (subscales pain + other symptoms) [59]	x	x	x	x			
Pain intensity	Numeric Rating Scale 0–10 for LBP and leg pain [64]	x	x	x	x			
Activity limitation	Oswestry Disability Index [62, 63]	x	x	x	x			
Quality of life	SF-36 general health, social functioning, mental health	x	x	x	x			
Work ability	Current work ability Number of days with LBP related sick leave within last 3 months	x	x	x	x			
Content of intervention	Received listed intervention yes/no		x					
Satisfaction	Overall are you satisfied with your course of care (5-point Likert scale)		x					
Harms	Did you experience any side effects or problems in relation to your participation in GLA:D Back?		x					
Back pain treatment	Consultations to general practice, chiropractors, physiotherapists and other clinicians due to back pain	x	x	x	x			
Health care utilisation	Reimbursed consultations to general practice, chiropractors and physiotherapists; Secondary care visits for back pain; Imaging for back pain Prescriptions for pain medication							x
Long-term sick leave	Number of reimbursed days with sick leave							x

##### Knowledge and skills

The Brief Illness Perception Questionnaire (B-IPQ) will be used for measuring illness beliefs [42, 43]. B-IPQ contains 9 nine items of which each covers one construct: consequences, timeline (expectations of prognosis), personal control, treatment control, identity (extent of symptoms), coherence (understanding of symptoms), emotional representation, concerns, and cause. Based on the properties of the scale observed in the pilot project, we expect to combine ‘consequences’, ‘identity’, ‘concerns’, and ‘emotional representation’ into ‘Interference’ (sum score 0–40), ‘personal control’ and ‘coherence’ into ‘Coherence and Control’ (sum score 0–20), and ‘timeline’ and ‘treatment control’ into ‘Expectations’ (sum score 0–20). If the internal consistency allows it, a sum score of items 1–8 will then be calculated as a measure of overall illness perceptions (0–80).

Perceived ability to perform exercises is measured by one question: ‘How confident are you in performing exercises in a beneficial way?’ (0–10 scale from ‘not confident at all’ to ‘absolutely confident’).

The four physical performance tests are: sit-to-stand test (number of repetitions in 30 s) [44, 45]; standing forward bending (0: No forward bending; 1: pain and restricted movement; 2: no pain and restricted movement; 3: pain and unrestricted movement; 4: no pain and unrestricted movement) [46–48]; iso-extensor endurance test (seconds in static position up till 3 min) [49, 50]; trunk flexor endurance test (seconds in static position up till 2 min) [50, 51]). All these tests are affected in chronic LBP, and the sit-to-stand test, extensor endurance and standing forward bending have been demonstrated to be responsive to change [52, 53].

##### Beliefs and performance

The B-IPQ ‘Expectations’ subscale measures recovery beliefs and the Fear Avoidance Beliefs Questionnaire physical activity scale (0 = no fear avoidance beliefs; 24 = highest possible fear avoidance beliefs) will be used to measure fear of movement [54–56].

Self-assessed physical fitness visual analogue scales compare perceived strength, endurance, flexibility, and balance with that of other people of the same age and sex and is used for measuring physical fitness [57, 58]. To capture the goal of moving freely we will add one item to self-assessed physical fitness and ask to what extent the patient perceives her−/himself ‘moving unhindered and naturally’.

##### Self-efficacy

The Arthritis Self-Efficacy Scale (ASES), subscales of pain and other symptoms will be used to assess symptom self-efficacy [59]. The ASES includes five items on pain and six on other symptoms. The scale was developed for arthritis but has also undergone validation also in a mixed group of patients with chronic pain [60] and in patients with fibromyalgia [61]. The questions ask about the degree of certainty that the respondent can manage with respect to aspects around pain, sleep, fatigue and mood. Each item is scored on a 0–10 scale (0 = very uncertain; 10 = very certain). For our purposes ‘arthritis’ will be changed to ‘back pain’.

##### Self-management success

The Oswestry Disability Index [62, 63], work ability (‘Imagine your ability to work is worth 10 points at its best. How many points will you give your current work ability?’ (0–10), LBP intensity (Numeric Rating Scale 0–10 [64]), quality of life (SF-36 subdomains of general health, social functioning, mental well-being), pain interference with life (B-IPQ ‘interference’), and achieved individual goal (SMART 0 = Not achieved at all - 10 = Fully achieved [40]) will be used to measure the success of self-managing pain. Because there is not one specific measure for this construct, other authors have suggested capturing a range of constructs including pain-related disability, pain intensity, quality of life, perception of social support, self-efficacy, pain acceptance, depression, anxiety, and general health as outcome measures of self-management interventions [65, 66].

##### System outcomes

Health care utilisation data (number of visits to GP, chiropractor and physiotherapist, prescription of pain medication, referrals to spine imaging, and referrals to secondary care for back pain) will be extracted from the Danish National Health Service Registry [67]. In addition, patients will be asked about care-seeking due to LBP and use of pain medication for LBP. Long-term sick leave (number of days off work after 1 month of absenteeism) is available in the ‘DREAM’ registry from the Ministry of Employment [68].

##### Patient characteristics

Age, sex, socio-economic status (education, job, income), LBP history (duration, recall of 1-year trajectory, previous care), and results from the Start Back Screening Tool (low, medium and high risk of poor prognosis) [69] will be collected at baseline in addition to the outcome measures mentioned above.

#### Outcomes of the clinician educational intervention

An overview of the study measures is provided in Table 5.

**Table 5:** Overview of study measurements related to the educational intervention targeted at clinicians

Construct	Instrument	Course registration	Before course	After course	5-months	Patient baseline registration	Patient reported at 3-months
Individual characteristics	Age, sex, profession	x					
Experience	Years of clinical experience Experience with GLA:D for knee and hip	x					
Clinic characteristics	Workplace (Private clinic, municipality, secondary care) Number and professional background of clinicians in the workplace	x					
Treatment orientation	The Pain Attitudes and Beliefs Scale for Physiotherapists [70–72]		x		x		
Confidence and capability	The Practitioner Confidence Scale [73] + 2 added items The Determinants of Implementation Behaviour Questionnaire (knowledge, skills, beliefs about capabilities) [74]		x		x		
Motivation	The Determinants of Implementation Behaviour Questionnaire (Beliefs about consequences, innovation, patients, intentions) [74]			x	x		
Opportunity	The Determinants of Implementation Behaviour Questionnaire (organisation, social influences, social context, and behavioural regulation) [74]			x	x		
Delivery of key messages	Patients asked to what extent they during the course of care they have heard about key messages of GLA:D Back (e.g. that pain does not signal harm) and messages contradictory to GLA:D Back (e.g. that certain positions or movements have to be avoided)						x
Establishing individual goals	SMART value-based goal registered at the initial consultation					x	

##### Outcomes related to learnings

The Pain Attitudes and Beliefs Scale for Physiotherapists (PABS-PT) is a self-administrated questionnaire developed to assess the strength of two possible treatment orientations of physiotherapists toward the management of back pain: predominantly biomedical orientation or predominantly behavioural orientation [70, 71]. We will use the 19-item version and added two questions based on a recent Rasch analysis of the Norwegian PABS (‘If ADL activities cause more back pain, this is not dangerous’; ‘Reduction of daily physical exertion is a significant factor in treating back pain’) [72]. The resulting biomedical subscale will consist of 11 items (sum score 11 to 66) and the behavioural subscale of 10 items (sum score 10 to 60).

The four-item Practitioner Confidence Scale (PCS) measures clinicians’ confidence in managing people with back pain [73]. Two items will be added to capture confidence about using a behavioral pain model (‘I feel confident using psychological and behavioural elements in the treatment of LBP patients’ and ‘I feel confident working with patient with LBP not basing this on a structural diagnosis’). Each item is scored on a five-point scale from 1 = ‘strongly agree’ to 5 = ‘strongly disagree‘, resulting in sum scores ranging from 6 to 30.

The Determinants of Implementation Behavior Questionnaire (DIBQ) will be used to measure the domains of knowledge, skills and capabilities about the delivery of GLA:D Back [74]. The DIBQ was developed from the Theoretical Domains Framework and covers 18 domains related to implementation processes [16]. We will include 31 items from the domains of knowledge (2 items), skills (1 items), beliefs about capability (6 items), beliefs about consequences (4 items), innovation (4 items), patients (2 items), intentions (1 item), organisation (2 items), social influences (3 items), social context (1 item), behavioural regulation (3 items), and innovation strategy (2 items). Items are scored 1 = strongly disagree to 7 = strongly agree, with negative statements reverse-coded.

##### Outcomes related to clinician behaviours

The delivery of a group-based patient education and exercise program will be assessed as the proportion of patients who reports participation in both of these activities. The delivery of key messages as part of the intervention will be measured as patients’ recall of having heard five key messages as part of their treatment course. Three of the listed messages are key messages in the GLA:D Back approach (‘Movement is healthy for the spine’, ‘Pain does not equal harm’, ‘Your brain has a memory for pain – like having a bad song stuck in your head’) and two are messages not in line with the intention of GLA:D Back (‘I need to avoid certain positions or movements’, ‘In my case, pain means I should stop what I am doing’). Both measures of delivery of care are included in the patient three-month follow up. Use of individual goals will be measured as the proportion of patients for whom a goal is described in the clinical registry.

#### Clinician and clinics characteristics

Information will be collected to describe clinician profiles: age, sex, profession, years of clinical experience, type of employment, experience with GLA:D knee and hip, and whether or not their clinical work is conducted under a contract with public health insurance. Information will also be collected about clinics: the number of physiotherapists, chiropractors, massage therapists and other clinicians in the clinic, whether GLA:D for knee and hip is offered, and if back pain group exercises were offered before introducing GLA:D Back.

#### Outcomes related to the national implementation

##### Promotion activities

Promotion of the course will be measured via the number of times GLA:D Back is mentioned in social media postings, newsletters and magazines (Table 2).

##### Reach, adoption and penetration

Clinician reach will be quantified as the number of clinicians signing up for the GLA:D Back courses, rate of adoption as the proportion of these who enrol patients in the program, and geographical dissemination as the extent of spread across the country (Table 3). The profiling of clinicians will include years of clinical experience, sex, profession (physiotherapist, chiropractor), place of employment (municipality, primary care clinic, secondary care), type of employment (clinic owner, employee, self-employed in a clinic owned by another person).

##### Fidelity

The outcome measures listed above about clinician behaviours are measures of fidelity.

##### Determinants of implementation

The DIBQ domains that relate to national implementation are organisation, social influences, social context, and behavioural regulation.

##### The profile of patients enrolled

Using data concerning the patient characteristics collected above, we will be able to profile patients enrolled in GLA:D Back.

##### Acceptability among patients

Clinicians will be asked ‘What proportion of invited patients would you say join GLA:D Back?’ (< 25%, 25–50%, approx. 50%, 50–75, > 75%), and ‘What are the main reasons why patients decline participation?’ (price, time point, don’t want supervised training, don’t want patient education, find the program too comprehensive, other reasons). If the last individual session does not occur, clinicians will report if the patient otherwise completed the intervention (Program completed without final session; Program not completed). Compliance with the program will be registered as the number of education sessions and exercise sessions patients report they attended when completing the three-months follow up.

##### Referrals and prescriptions

Suitable outcomes from national registries and population definitions for a pre-post comparison at the national level are still to be determined and will be informed by the characteristics of the population enrolled in GLA:D Back. Available information includes reimbursed visits to general practice, physiotherapists, and chiropractors, visits and diagnostic codes from hospital visits, referrals to reimbursed imaging and prescriptions of medication.

### Sample size

The study population will be the clinicians who complete GLA:D Back courses during 2018 and the patients enrolled in GLA:D Back by these clinicians during a 6-months window of inclusion commencing after a 3-month phase of familiarisation with the program after the course. Assuming that clinicians from 150 clinics will participate in the GLA:D Back course during 2018, and they enrol 1500 patients during the study period, we will have 90% power to demonstrate pre-post comparison changes at the patient level with an effect size of 0.2 while accounting for clustering within clinics and clinicians up to a variance inflation factor of 5. At the clinician level, we will have [75]90% power to demonstrate changes with an effect size of 0.27.

### Statistical analyses and reporting

Due to the wide scope of the study, analyses and reporting will be conducted in several dedicated subprojects. These will be organized along the main hypotheses, the main topics mentioned under “Effectiveness and process evaluations ”, and the time frame of the study. A detailed analysis plan and a publication and dissemination plan will be prepared for each subproject. An initial data analysis will be performed according to the recommendations of the STRATOS (STRengthening Analytical Thinking for Observational Studies) initiative [75], informing all subprojects about the available meta data, the patient flow in the project and the distribution of key variables. Across all sub projects we will make use of hierarchical models to take the hierarchical structure (clinics and clinicians within clinics) into account. Latent class models will be used to detect patient and clinician profiles and structural equation models will be used to investigate the associations among latent variables. Mediation analysis will be performed according to the causal framework described by Vanderweele [76]. The overall research team takes the responsibility to prepare an additional publication summarizing the results of the different subprojects.

## Discussion

This implementation-effectiveness study is designed to evaluate the process and outcomes of implementing a standardised intervention, GLA:D Back, for people with persistent or recurrent LBP. The implementation strategy targets clinicians and the clinical intervention targets people seeking care for persistent or recurrent LBP who are in need of improved self-management. In this study, we will investigate how standardised evidence-based care for LBP can become widely available, how the intervention will be delivered and to whom, what outcomes patients achieve in clinical practice, and what the effects will be at the national level. Further, we investigate potential mechanisms for effects by evaluating changes in several intermediate outcomes based on theoretical models for introducing behaviour changes and for supporting self-management of back pain.

The implementation strategy with a 2-day course for clinicians mirrors that of the GLA:D program for patients with knee and hip pain [31]. That program has achieved very large national adoption and has developed into an international model of standardised care with training of clinicians in Canada, Australia and China within only 5 years of its commencement [77, 78]. Organisations and clinicians have subsequently called for a similar model for back pain care and thus, GLA:D for knee and hip pain serves as a model for the implementation of evidence-based back pain care. A pilot study testing the feasibility of GLA:D Back showed good promise for implementation (nine test sites all enrolled patients in the GLA:D Back program within 2 months of clinicians’ course participation and clinicians’ and patients’ perceptions were positive) (manuscript in preparation).

Despite good intentions, implementation of health care initiatives is complex, a lot of factors affect implementation and very many aspects deserve evaluation. Therefore, implementation is best understood within a theoretical framework and should be planned and evaluated within one [79]. We will use the Behavior Change Wheel, which describes interventions and policies related to change of behaviour [35]. This framework facilitates a focused evaluation and also clarifies aspects of supporting behavioural change that our implementation strategy does not include. For example, we did not involve decisions-makers in health care from the beginning of the project and are aware that the existing reimbursement structure may be an important barrier to implementation.

This study initiates a line of research with the potential to generate insight into real life effects of recommended treatments for people with persistent or recurrent LBP, and into ways to assist clinicians in delivering this care. The clinical registry will continue after the study period and will generate a cohort for future monitoring of outcomes, as well as for embedded trials for testing alterations to the education of clinicians and to the clinical intervention. Educational interventions for clinicians using a workshop format with didactic and interactive elements have been used for implementation of guidelines on back pain [80] and osteoarthritis [81], but the amount and type of training needed for clinicians to deliver a new intervention with satisfactory fidelity is unknown. Thus, the effects of a more comprehensive training of clinicians is one aspect that can be tested in a future embedded trial. Also, the investigations of intermediate outcomes may inform alterations to the clinical intervention that can then be tested for effectiveness in a nested study.

Some compromises were made in the design of this study. First, given the large evidence base for patient education and exercise, we did not aim at a further evaluation of the clinical intervention in a randomised design. Instead, we focused on the implementation of such an intervention and an investigation into how delivery of care can be optimised in clinical practice. However, also an evaluation of the educational intervention for clinicians or the overall implementation could also have been undertaken in a cluster-randomised design. Unfortunately, due to the nationwide spread of the existing GLA:D knee and hip program and the high expectations associated with its extension to low back pain patients, a randomisation at the level of clinics or regions would not have met wide acceptance, and we did not adopt that design. Pragmatically, we investigate the effect of the clinical intervention itself only by a pre-post comparison among the participating patients. We originally planned to include at least a pre-post comparison based on two cross-sectional samples drawn from each clinic prior to, and after, introducing GLA:D Back. However, as the intervention will also change the composition of the patient population approaching the clinics, it was not possible to define a relevant patient group that would have resulted in comparable pre and post populations.

Another challenge in evaluating self-management interventions is the choice of outcomes. Of course, for long-term outcomes at the patient and societal levels, we could use established instruments and indicators. However, our aim is also to understand the intermediate process in reaching a long-term improvement, i.e. how a better self-management is reached and how it affects the long-term outcomes. The construct of self-efficacy is relatively well-defined and related measurement tools exist. More challenging is the measuring of self-management. It is not clear what defines the ability to self-manage and a large number of different tools and constructs have been used for measuring aspects of this [66]. As part of this study, we will investigate mechanisms behind the development of self-efficacy and self-management by testing hypothesised pathways and by combining quantitative and qualitative data to improve our understanding of patients’ perceptions of self-management.

In conclusion, this hybrid implementation-effectiveness study will evaluate efforts to implement a program aimed at promoting self-management of persistent and recurrent back pain in people presenting in Danish primary care and demonstrate what the actual target group is for this type of care and the outcomes achieved. This will help to develop strategies for implementing evidence-based care for back pain and potentially other musculoskeletal pain conditions.

### Organisation

The study is conducted by University of Southern Denmark, Department of Sports Science and Clinical Biomechanics. An advisory board is informed about study plans and progress and provides input but has no authority over project activities. University of Southern Denmark holds the GLA:D® trademark.

## Additional files

Additional file 1: Plan for the two-day course. (DOCX 20 kb)

Additional file 2: English translation of patient consent. (PDF 107 kb)

## Abbreviations

ASES: Arthritis Self-Efficacy Scale
BCW: Behaviour Change Wheel
B-IPQ: Brief Illness Perceptions Questionnaire
DIBQ: Determinants of Implementation Behaviour Questionnaire
DPA: Danish Data Protection Agency
GLA:D: Good Life with osteoArthritis in Denmark (only the acronym is used in GLA:D Back)
GP: General Practitioner
LBP: Low back pain
OPEN: Odense Patient data Explorative Network
PABS: Pain Attitudes and Beliefs Scale
PCS: Practitioner Confidence Scale
RCT: Randomised Controlled Trial
