# Supplementary material for: GLA:D® Back: implementation of group-based patient education integrated with exercises to support self-management of back pain - protocol for a hybrid effectiveness-implementation study
Source: BMC Musculoskelet Disord. 2019 Feb 18;20:85. doi: 10.1186/s12891-019-2443-1 (PMC6380042; doi:10.1186/s12891-019-2443-1)
Supplement: Supplementary file 1 — Plan for the two-day course. (DOCX 20 kb) [file 12891_2019_2443_MOESM1_ESM.docx]

| **DAY 1** | |
| --- | --- |
| 8.30-9.00  9.00 | **Arrival**  **Welcome**  **‘State of the art’ - News and updates on back pain** |
|  | **GLA:D Back – Background and principles** |
| 10.30 | BREAK |
| 10.45 | **Overview of the GLA:D Back program** **Patient education –** Key messages |
| 11.45 | LUNCH |
| 12.45 | **Group exercise: Delivering key messages of the patient education** |
| 14.15 | BREAK |
| 14.30 | **The GLA:D Back clinical registry** – Why and how?  **SMART goal setting** |
| 15.30  16.00 | **Summary of the main points of today**  **Close** |

| **DAG 2** | |
| --- | --- |
| 8.30-9.00  9.00 | **Arrival**  **Clinical tests (theory)** |
| 9.30 | **Exercise therapy (theory)** – Content of the program and format |
| 10.10 | Break |
| 10.30 | **Clinical tests (practical)** |
| 11.30 | LUNCH |
| 12.00 | **Exercises (practical)** |
| 13.15 | BREAK |
| 13.30  14.20 | **Group exercise: Instructions of exercises**  BREAK |
| 14.35 | **Organisation and plan** – How to get started |
| 15.30  16.00 | **Summary and plenary discussion of barriers**  **End** |
