# Supplementary material for: GLA:D® Back: implementation of group-based patient education integrated with exercises to support self-management of back pain - protocol for a hybrid effectiveness-implementation study
Source: BMC Musculoskelet Disord. 2019 Feb 18;20:85. doi: 10.1186/s12891-019-2443-1 (PMC6380042; doi:10.1186/s12891-019-2443-1)
Supplement: Supplementary file 2 — English translation of patient consent. (PDF 107 kb) [file 12891_2019_2443_MOESM2_ESM.pdf]

GLA:D BACK – Letter of consent

Velkommen til dit GLA:D Ryg-forløb.

Formål

Som led i forløbet indsamler forskere fra Syddansk Universitet oplysninger om deltagerne. Formålet er at undersøge effekten af GLA:D Ryg, og finde ud af hvem der har størst gavn af tilbuddet og hvorfor.
Ved at besvare spørgeskemaet her og igen om 3, 6 og 12 måneder bidrager du til at forbedre behandlingen for rygsmerter. Jo flere der deltager - uanset hvilken grad af rygbesvær man har - jo mere sikre resultater får vi.

Hvilke oplysninger indsamler vi

- Vi indsamler helbredsoplysninger, der relaterer sig til rygproblemer, herunder smerte, funktion og dit generelle helbred. Der indsamles desuden information om arbejde og sygemelding.
- Dine oplysninger anvendes kun til at udvikle og sikre behandlingens kvalitet, fremstille statistik samt til forskning indenfor sundhedsvæsenet.
- Til dele af vores forskning vil vi indhente oplysninger fra registre, fx om du har været sygemeldt eller har været hos læge eller på hospitalet. Vi får registeroplysninger på alle deltagere på én gang i anonymiseret form.

Hvad indebærer din deltagelse

- Du svarer på spørgemålene, der følger på de næste sider.
- Du får yderligere 3 spørgeskemaer inden for det næste år.
- Den behandling, du får, påvirkes *ikke* af din medvirken.

Hvordan behandles dine oplysninger

- Data videregives kun til rygforskere fra SDU og deres samarbejdspartnere.
- Alle oplysninger bliver anonymiseret, så det ikke fremgår af resultaterne, hvem der har bidraget til registret. Din kiropraktor/fysioterapeut får heller ikke at vide hvad du svarer.
- Oplysningerne opbevares, så længe de indgår i GLA:D Rygs forskningsprojekter.

Dine rettigheder

- Deltagelse er frivillig, og du kan til enhver tid stoppe din deltagelse, hvis du ønsker det.
- Du har ret til indsigt i de oplysninger vi opbevarer og behandler om dig.
- Desuden har du ret til at gøre indsigelse mod databehandlingen, til at få urigtige oplysninger rettet eller slettet, få begrænset behandlingen af dine oplysninger, samt til at få egne data udleveret.

Welcome to your GLA:D Back program.

Purpose

As part of the program, researchers from the University of Southern Denmark collect information on the participants. The purpose is to investigate the effect of GLA:D Back and find out who benefits the most of the program and why.
By answering this questionnaire and the follow-ups at 3, 6 and 12 months you help to improve the treatment for back pain. The more participants we get - no matter what degree of back problems you may have - the more precise results.

What information do we collect

- We collect health information that relates to back problems, including pain, function and your overall health. Information on work and sick leave is also collected.
- Your information is solely used to develop and ensure the quality of the treatment, to produce statistics and to research in the field of health science.
- For parts of our research we will retrieve information from registers, e.g. if you have been off work, hospitalized or have seen your GP. We obtain registry information on all participants at once in anonymous format.

What will your participation imply?

- You will answer the questions on the following pages.
- You will receive another 3 questionnaires within the next year.
- The treatment you receive is *not* affected by your participation.

How is your information processed?

- Data is only disclosed to back researchers from SDU and their collaborators.
- All information is anonymized, so it is not clear from the results who contributed to the registry. Your chiropractor / physiotherapist will not know your response.
- The information is stored as long as they are part of GLA:D Back's research projects.

Your rights

- Participation is voluntary and you can stop your it at any time you wish.
- You have a right of insight into the information on you we store and process.
- In addition, you have the right to object to data processing, to have incorrect information corrected or deleted, to have limited processing of your information, as well as to have your own data provided.

Klageadgang<br>Du kan klage over behandlingen af dine personoplysninger til Datatilsynet, Borgergade 28, 1300 København K, telefonnummer 33 19 32 00, eller via e-mail: dt@datatilsynet.dk<br>Du kan også altid kontakte SDU's databeskyttelsesrådgiver på dpo@sdu.dk<br><br>Dataansvarlig og kontaktperson<br>Syddansk Universitet er dataansvarlig for behandlingen af personoplysninger.<br><br>Kontaktperson er projektleder Alice Kongsted, lektor Institut for Idræt og Klinisk Biomekanik, Syddansk Universitet<br><br>Du kan kontakte os via mail til: gladryg@sdu.dk	Right to complain<br>You may complain about the processing of your personal data to Datatilsynet, Borgergade 28, 1300 Copenhagen K, phone number 33 19 32 00, or by e-mail: dt@datatilsynet.dk<br>You can also always contact SDU's Data Protection Advisor at dpo@sdu.dk<br><br>Data management and contact person<br>The University of Southern Denmark is the data controller for the processing of personal data.<br><br>Contact person is project manager Alice Kongsted, Associate Professor<br>Department of Sport and Clinical Biomechanics, University of Southern Denmark<br><br>You can contact us by mail to: gladryg@sdu.dk
